# A Scene-Adaptive Super-Resolution Framework for Video Compression

**DOI:** 10.3390/jimaging12050200

**Published:** 2026-05-05

**Authors:** Qiyu Zha, Jiangling Guo

**Affiliations:** College of Intelligent Science and Engineering, Jinan University, 206 Qianshan Road, Xiangzhou District, Zhuhai 519000, China; zhaqiyu99@stu2023.jnu.edu.cn

**Keywords:** video compression, super-resolution, scene-adaptive segmentation, parameter-efficient fine-tuning, adapter, chained differential coding

## Abstract

Video compression is central to large-scale video delivery, where better rate–distortion efficiency directly reduces bandwidth and storage cost. A practical way to improve efficiency is to encode a low-resolution video stream with a standard codec and restore high-resolution details with a learned super-resolution model at the decoder. However, prior SR-assisted compression methods usually update the reconstruction model at fixed temporal intervals, which can waste bitrate when those update boundaries do not match actual scene changes. In this paper, we present SASVC, a scene-adaptive super-resolution video compression framework for offline codec-augmented compression. SASVC detects scene changes using frame-wise grayscale differences, updates only compact adapter modules when a content transition is observed, and compresses the resulting model updates with chained differencing, quantization, and entropy coding. In this way, the method reduces unnecessary model-stream overhead while preserving scene-specific reconstruction fidelity. Experimental results on both long-form and short-form datasets show that SASVC consistently outperforms SRVC-style baselines and conventional codec-based alternatives under the Bjontegaard delta rate based on peak signal-to-noise ratio (BD-rate/PSNR) criterion. Complementary rate–distortion (RD) comparisons in terms of structural similarity index measure (SSIM) and Video Multi-Method Assessment Fusion (VMAF) show the same overall trend, indicating that the gain is not limited to a single distortion metric. Specifically, SASVC achieves BD-rate gains of −41.33% and −53.49% on Vimeo and Xiph, respectively, and further reaches −51.53% and −39.83% on UVG and MCL-JCV. The decoder also maintains real-time 1080p reconstruction at 125 frames per second (FPS) on an NVIDIA RTX 3080 Ti GPU, indicating that scene-aligned model updates can improve compression efficiency while keeping decoder-side deployment practical.

## 1. Introduction

Video traffic continues to grow at an unprecedented rate, placing increasing pressure on network bandwidth and storage systems [1,2]. As high-resolution formats such as 1080p and 4K become increasingly common, the cost of video delivery rises accordingly. Improving rate–distortion efficiency without introducing prohibitive computational overhead, therefore, remains a central challenge in modern video compression [1,2].

Despite this demand, most practical video codecs still rely on principles developed decades ago, including transform coding and motion estimation [3,4,5,6]. While standards such as H.265/High Efficiency Video Coding (HEVC) improve coding efficiency through extensive engineering refinements [4], their reconstruction behavior remains fixed once the bitstream is formed. Recent deep learning-based video compression methods attempt to learn larger parts of the coding pipeline [7,8,9,10], and some newer systems have improved runtime compared with early neural codecs. Even so, end-to-end learned compression remains substantially harder to deploy than standard codecs in practical high-resolution pipelines because it often requires specialized neural encoding/decoding stacks, heavier inference, and less mature ecosystem support [7,8,11].

A practical alternative is to retain a standard codec for the content stream and apply a learned super-resolution (SR) model only at reconstruction time [12,13,14,15]. This codec-augmented design shifts part of the bitrate budget from explicit HR pixels to model parameters while preserving compatibility with mature video-delivery infrastructure. However, existing SR-assisted compression methods such as SRVC [16] typically divide videos into fixed-length segments and periodically update a small subset of parameters. Such uniform temporal partitioning does not reflect actual scene changes and can introduce unnecessary model-stream overhead.

To address these limitations, we propose **SASVC** (scene-adaptive super-resolution video compression), a scene-adaptive SR-assisted compression framework that better balances adaptation accuracy and model-stream overhead. SASVC consists of three stages. First, it performs lightweight scene-adaptive analysis using frame-wise grayscale differences to partition videos according to content dynamics rather than fixed temporal intervals. Second, it applies adapter-based parameter-efficient fine-tuning (PEFT) [17,18] to specialize a lightweight SR backbone for each detected segment. Third, it compresses the scene-specific adapter parameters using chained differential coding, quantization, and entropy coding [19,20,21,22,23].

Compared with SRVC-style approaches, the main difference in SASVC is not only that model updates are triggered by scene-aligned boundaries, but also that the updates are confined to compact adapter modules instead of directly modifying selected backbone weights [16]. This reduces the dimensionality of the transmitted update, improves control over model-stream cost, and makes scene-wise specialization more practical under tight bitrate budgets. In addition, by exploiting redundancy across adjacent scene segments through chained parameter differencing, SASVC progressively compresses model updates over time.

A key observation behind our design is that in many practical video delivery scenarios, the content is available offline before encoding [16]. In this setting, broad generalization across arbitrary inputs is less important than content-specific specialization, and encoder-side optimization cost can be amortized over repeated playback or distribution. We therefore target offline or pre-distribution settings such as video-on-demand and archival delivery rather than strict live streaming; this scope is an intentional design choice and also a practical limitation of the current method.

In summary, our contributions are threefold:

- We propose a lightweight scene-adaptive update scheduler for SR-assisted video compression, where frame-wise grayscale differences are used to trigger model updates according to content transitions rather than uniform temporal windows.
- We replace direct sparse backbone updates with adapter-based scene specialization and combine it with chained differential coding, yielding a more compact and controllable model stream than prior SRVC-style designs.
- We demonstrate that this scene-aligned specialization strategy improves rate–distortion performance across both long-form and short-form datasets while preserving real-time decoder-side reconstruction efficiency.

## 2. Related Work

Standard codecs. Conventional video codecs, including H.264/AVC, H.265/HEVC, VP9, and AV1, remain the dominant solutions in practical video delivery systems [3,4,5,6]. These standards rely on carefully engineered components such as transform coding, motion estimation, motion compensation, and entropy coding to exploit spatial and temporal redundancy in video signals. Although modern codecs improve coding efficiency through refinements such as variable block partitioning and improved in-loop filtering, their core design remains largely fixed and cannot adapt reconstruction behavior to the content of a specific video [3,4]. Existing codecs are especially strong in offline high-quality settings, where slower encoding modes can further improve compression efficiency. Nevertheless, recent SR-assisted compression frameworks indicate that combining a low-resolution coded stream with a learned reconstruction model can outperform direct high-resolution coding by a standard codec alone [16]. Some practical codecs also support low-resolution coding followed by interpolation-based upsampling, but learned reconstruction generally yields larger quality improvements than fixed interpolation kernels [6,24].

Super-resolution for compressed video. Single-image and video super-resolution have made substantial progress with deep neural architectures, consistently surpassing classical interpolation methods such as bilinear and bicubic upsampling [12,13,14,15,24]. To control computation at high output resolutions, many efficient SR models keep most feature processing in low-resolution space and postpone spatial expansion to the final stage through efficient upsampling operators [25]. Multi-frame video SR can further improve reconstruction by exploiting temporal information across neighboring frames [26,27,28]. Beyond conventional recurrent CNN designs, recent video restoration work has also explored transformer-based or hybrid architectures such as RVRT and VRT, which can further improve restoration quality at the cost of higher memory or runtime demand [29,30]. In addition, compressed-video-aware restoration methods such as COMISR and Compression-Aware Video Super-Resolution explicitly account for codec artifacts and compression levels, making them more relevant than generic bicubic-only video super-resolution (VSR) baselines when discussing codec-augmented reconstruction [31,32]. In contrast, our target setting is decoder-side reconstruction inside an SR-assisted compression pipeline, where efficiency and controllable model-stream size are as important as generic restoration accuracy. Compared with prior SR-assisted compression approaches, especially SRVC [16], SASVC further improves the adaptation process by replacing fixed-length temporal partitioning with scene-adaptive segmentation and by isolating the video-specific update inside lightweight adapters.

Learned video compression. End-to-end learned video compression methods replace parts of the conventional coding pipeline with neural modules, such as learned motion estimation, motion compensation, and latent-space residual coding [7,8,9,10,11]. More recent conditional-coding systems, including DCVC and its follow-up variants, have substantially improved coding efficiency and practical speed relative to early neural codecs, making the comparison with conventional codecs more nuanced than before [33,34,35]. Nevertheless, deployment remains challenging in high-resolution and throughput-sensitive settings because learned codecs generally require heavier neural computation and a fully learned decoding stack. By contrast, SR-assisted codec augmentation follows a different design philosophy: instead of replacing the entire codec, it retains a standard codec for low-resolution content transmission and applies a learned restoration model only at the reconstruction stage. This hybrid strategy better preserves compatibility with mature codec infrastructure while still benefiting from content-adaptive neural enhancement, with SRVC being the most directly related prior work in this design space [16].

Parameter-efficient adaptation and model-side compression. Recent work on parameter-efficient fine-tuning (PEFT), including adapter modules and low-rank adaptation, has shown that compact task-specific components can effectively specialize pretrained networks while updating only a small fraction of parameters [17,18]. In SR-assisted video compression, this choice is especially important because any trainable parameter may become part of the transmitted bitstream. We adopt bottleneck adapters because they localize scene-specific updates into explicit modules with clear parameter budgets, making the model-stream size easier to control. Compared with broader low-rank modification or prompt-style conditioning, adapters provide a straightforward trade-off between expressivity, modularity, and transmission cost for codec-augmented SR compression. Compared with prior SR-based compression methods that directly update selected backbone parameters [16], SASVC confines video-specific adaptation to lightweight adapter modules, substantially reducing update dimensionality and simplifying per-segment specialization. In parallel, model compression techniques such as pruning, quantization, entropy coding, and learned compression have long been studied for reducing storage and deployment cost [19,20,21,22,23]. In our setting, these techniques become directly relevant because the adapted weights themselves must be transmitted together with the compressed video. Beyond applying standard compression tools, SASVC explicitly exploits temporal redundancy across adjacent scene segments through chained differential coding, followed by quantization and entropy coding, to more aggressively reduce model-stream overhead.

## 3. Materials and Methods

**Figure 1:**
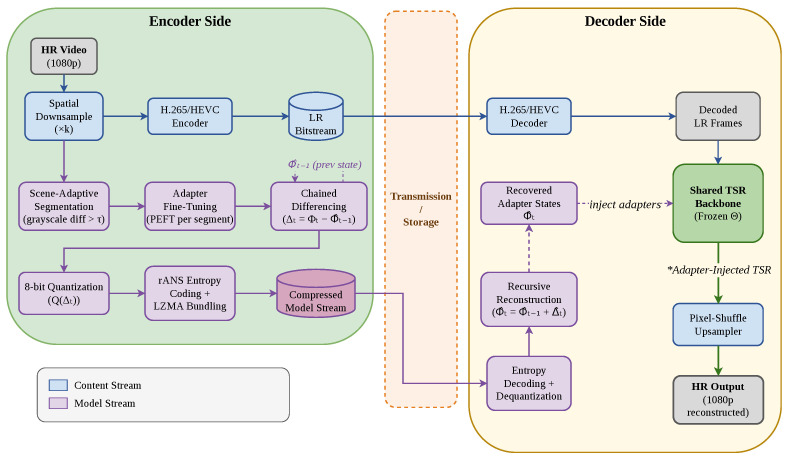
Overview of the proposed scene-adaptive super-resolution video compression (SASVC) framework. Blue elements denote the content stream, which carries the encoded low-resolution (LR) video through a standard H.265/High Efficiency Video Coding (HEVC) codec. Purple elements denote the model stream, which carries compressed scene-specific adapter states derived from scene-adaptive segmentation and per-segment specialization. The decoder combines the reconstructed LR bitstream and the recovered adapter states in a shared TSR backbone to produce the final high-resolution (HR) output.

Figure 1 illustrates the overall pipeline of **SASVC**. Similar to prior super-resolution-assisted compression frameworks such as SRVC [16], SASVC encodes each input video into two coordinated bitstreams: a content stream and a model stream. The central difference is that our model stream is constructed through scene-adaptive partitioning, adapter-based parameter-efficient specialization, and structured weight compression, rather than through fixed-length segmentation and direct sparse backbone updates [16].

### 3.1. Overall Framework

Content stream. Given an input high-resolution (HR) video, the encoder first downsamples each frame by a spatial factor of *k* in each dimension to obtain low-resolution (LR) frames. This design follows the general principle of codec-augmented SR-based compression, where part of the bitrate budget is shifted from explicit HR pixel transmission to a learned reconstruction model [12,14,16]. The LR frames are then encoded using an off-the-shelf video codec. In our implementation, we use H.265/HEVC [4]. At the decoder, the same codec reconstructs the LR frames. Since practical codecs are lossy, the decoded LR frames do not exactly match the encoder-side downsampled frames.

Model stream. A second bitstream encodes scene-specific adapter parameters used by the decoder to enhance the reconstructed LR frames. Instead of dividing the video into fixed-length temporal segments as in prior work [16], SASVC first partitions the input into scene-adaptive chunks according to frame-to-frame content variation. For each scene segment t∈{0,…,N−1}, the encoder specializes a lightweight adapter attached to a shared SR backbone using the decoded LR frames from that segment as input and the original HR frames as supervision. Let $\Phi_{t}$ denote the adapter parameters for segment *t*. The model stream starts from an initial adapter state and then transmits compressed updates across segments. At the decoder, the backbone remains fixed, while the adapter state is updated segment by segment and used to reconstruct HR frames from the decoded LR input.

Compared with transmitting a full model for each segment, this design significantly reduces model-stream overhead by restricting adaptation to compact adapter modules and explicitly compressing their temporal variation. This separation between a codec-based content stream and a compressed adaptive model stream also preserves the robustness of conventional codecs while enabling content-specific neural enhancement [4,16].

Figure 1 can therefore be read as a dual-stream pipeline: the content stream carries the conventional codec-compressed LR video, whereas the model stream carries only the scene-specific adapter updates needed to specialize the fixed backbone. This distinction is important because SASVC improves efficiency not by replacing the codec, but by coordinating a mature codec pipeline with a compact scene-aligned neural side stream.

### 3.2. Scene-Adaptive Segmentation

Unlike prior methods that rely on fixed-length segmentation [16], SASVC detects scene boundaries using frame-wise grayscale differences computed directly from the decoded video stream. This choice is motivated by the observation that abrupt changes in luminance structure often correspond to scene transitions or substantial content shifts, which in turn imply that the SR model should be updated.

Given two consecutive frames, we convert them to grayscale and compute the mean absolute difference:

(1) $$d_{i}=\frac{1}{HW}\sum_{u=1}^{H}\sum_{v=1}^{W}\left|G_{i}\left(u,v\right)-G_{i-1}\left(u,v\right)\right|,$$

where $G_{i}$ and $G_{i-1}$ denote the grayscale versions of the *i*-th and (i−1)-th frames, respectively, and H×W is the frame resolution.

A new scene segment is started whenever

(2) $$d_{i}>\tau ,$$

where τ is a threshold controlling the trade-off between over-segmentation and under-segmentation. In the main experiments, we keep τ fixed across datasets rather than tuning it per sequence, so that the same segmentation rule is applied throughout the evaluation. In our implementation, τ is set on the 8-bit grayscale-difference scale to provide a practical balance: smaller values trigger too many short segments and increase model-stream overhead, while larger values merge visually different content and reduce the benefit of scene-specific adaptation. This produces a sequence of chunk ranges

(3) $$S=\{\left[s_{0},e_{0}\right],\left[s_{1},e_{1}\right],\ldots ,\left[s_{N-1},e_{N-1}\right]\},$$

which are then used as the unit of model adaptation.

Compared with fixed temporal windows, this content-driven strategy better aligns parameter updates with actual scene transitions, reducing redundant updates across visually stable regions while allowing additional flexibility at abrupt content changes. In practice, this is especially useful for videos with irregular pacing, where fixed-length segmentation may split a coherent scene into multiple segments or merge visually distinct scenes into a single update interval [16]. The rule is intentionally lightweight: it is designed as an update scheduler rather than as a full semantic scene detector. In the current implementation, we deliberately avoid extra temporal smoothing so that the segmentation signal remains simple, transparent, and inexpensive, but this also means that the detector may be less reliable under fast camera motion, flashing lights, or gradual transitions. In such cases, stronger motion-aware analysis, temporal filtering, or learned scene analysis could further improve boundary detection. We discuss this limitation again in Section 5.

### 3.3. TSR Backbone and Adapter-Based Scene Specialization

**Shared SR backbone.** For reconstruction, SASVC adopts *TSR* as the shared SR backbone. TSR is an efficient super-resolution architecture developed in the context of the NTIRE 2025 efficient super-resolution challenge [36]. We choose TSR because it offers a strong efficiency–accuracy trade-off for codec-augmented decoder-side reconstruction: most feature processing is performed in low-resolution space, the architecture remains lightweight enough for real-time deployment, and the frozen backbone provides a stable generic reconstructor on top of which scene-specific specialization can be added. In our implementation, the backbone consists of an input convolution layer, a stack of feature transformation blocks, a reconstruction convolution layer, and a final pixel-shuffle upsampler [25]. This design follows the widely used strategy of performing most feature processing in low-resolution space and deferring spatial expansion to the final stage, which is known to reduce inference cost while preserving reconstruction quality [14,25,26].

Let $g_{\Theta }\left(\cdot \right)$ denote the frozen TSR backbone with parameters Θ. Given an LR input frame *x*, the backbone computes

(4) $$\hat{y}=g_{\Theta }\left(x\right),$$

where the final spatial upsampling is performed by a sub-pixel rearrangement module [25]. A residual skip from the shallow features to the reconstruction stage is retained to stabilize optimization and preserve low-frequency content.

**Adapter insertion.** To enable scene-specific specialization without updating the full backbone, we insert lightweight bottleneck adapters after each intermediate feature block and after the penultimate reconstruction convolution. This follows the general principle of parameter-efficient fine-tuning (PEFT), where compact trainable modules are used to adapt a largely frozen pretrained model while updating only a small subset of parameters [17,18].

**Figure 2:**
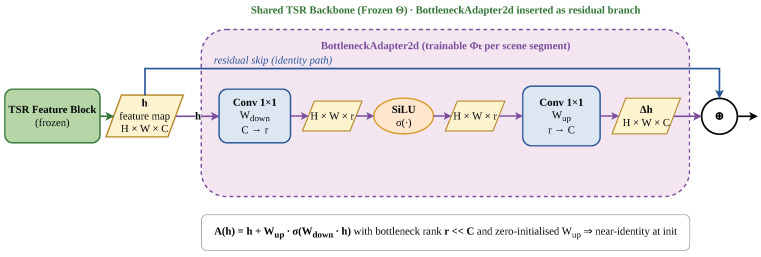
Structure of the BottleneckAdapter2d module in the revised left-to-right layout. The adapter projects the feature map from *C* channels to a low-rank bottleneck of size *r*, applies a SiLU non-linearity, and projects it back to *C* channels. The resulting residual is added to the input feature map, while the shared TSR backbone remains frozen.

Let *h* denote an intermediate feature tensor with *C* channels. Each adapter computes a residual correction of the form

(5) $$A\left(h\right)=h+W_{\mathrm{up}}\;\sigma \;\left(W_{\mathrm{down}}h\right),$$

where $W_{\mathrm{down}}$ and $W_{\mathrm{up}}$ are 1×1 convolutional projections, σ(·) is a non-linear activation, and the bottleneck rank is much smaller than *C*. This bottleneck design reduces the number of trainable parameters while preserving the expressive ability needed for scene-specific residual refinement. In our implementation, the up-projection is initialized to zero so that each adapter starts from a near-identity mapping, which makes training stable and ensures that the insertion of adapters does not perturb the frozen backbone at initialization. We prefer adapters over broader low-rank or prompt-style conditioning in this setting because the adapted parameters themselves must be transmitted: bottleneck adapters make the update locations explicit, keep the parameter budget easy to bound, and integrate naturally with a frozen convolutional backbone. The adapter structure is illustrated in Figure 2.

Let $\Phi_{t}$ denote the adapter parameters for segment *t*. The scene-conditioned SR model is written as

(6) $$\hat{y}=g_{\Theta ,\Phi_{t}}\left(x\right),$$

where Θ is frozen and only $\Phi_{t}$ is optimized for the current scene.

**Training objective.** For each scene segment $S_{t}$, the adapter parameters are optimized using the luminance-channel reconstruction loss:

(7) $$L\left(\Phi_{t}\right)=\frac{1}{|S_{t}|}\sum_{j\in S_{t}}{∥{\hat{Y}}_{j}-Y_{j}∥}_{1},$$

where $Y_{j}$ is the ground-truth HR luminance frame and ${\hat{Y}}_{j}$ is the predicted luminance output for frame *j*. We use only the Y channel because luminance dominates perceptual sharpness and is the standard optimization target in many super-resolution and compression pipelines [12,13,14]. In the final codec-based reconstruction pipeline, the chroma channels follow the decoded content stream and are combined with the super-resolved luminance output for evaluation in the YUV domain. This choice keeps the training target focused on the component that most strongly affects perceived detail while remaining consistent with standard compression-oriented evaluation.

The adaptation is performed sequentially across segments:

(8) $$\Phi_{t}\leftarrow \mathrm{Train}\left(S_{t};\Phi_{t-1}\right),$$

that is, the adapter for the current segment is initialized from the previous segment’s adapter state. This warm-start strategy improves convergence, encourages temporal consistency between adjacent scenes, and increases parameter correlation across segments, which is also beneficial for subsequent weight compression.

### 3.4. Model-Side Compression

Although adapter parameters are much smaller than full-model updates, transmitting them independently for every segment still introduces non-negligible overhead. To reduce this cost, SASVC compresses the model stream in three stages: chained differencing, quantization, and entropy coding. These stages are inspired by classical model-compression and data-compression strategies, but are adapted here to the sequential, scene-conditioned nature of the model stream [19,20,21,22,23].

**Chained differential coding.** Let $\Phi_{t}$ denote the adapter parameters of segment *t*. Instead of transmitting $\Phi_{t}$ directly, SASVC encodes the difference between adjacent adapter states:

(9) $$\Delta_{t}=\Phi_{t}-{\hat{\Phi }}_{t-1},$$

where ${\hat{\Phi }}_{t-1}$ is the *reconstructed* adapter state from the previous segment. Using the reconstructed state as the predictor ensures encoder–decoder consistency in the presence of quantization. The decoder then recursively reconstructs the current adapter state as

(10) $${\hat{\Phi }}_{t}={\hat{\Phi }}_{t-1}+{\hat{\Delta }}_{t}.$$

Because adjacent scene segments often share similar content and because the adapters are optimized sequentially, $\Delta_{t}$ typically has a smaller dynamic range than the raw parameters, which improves compressibility.

**Quantization.** To further reduce bitrate, the chained differences are quantized before transmission. In our implementation, we use fixed 8-bit quantization:

(11) $$Q\left(\Delta_{t}\right)=\mathrm{round}\;\left(\frac{\Delta_{t}}{s}\right),$$

where *s* is the quantization step size determined by the quantizer configuration. The decoder applies the corresponding inverse scaling to obtain ${\hat{\Delta }}_{t}$. We use fixed 8-bit quantization because it provides a practical operating point in our setting: lower precision noticeably degrades reconstructed adapter states, while higher precision yields limited additional RD benefit relative to the larger transmitted payload.

**Entropy coding and bundling.** The quantized symbols are then compressed using range Asymmetric Numeral Systems (rANSs), implemented through a categorical entropy model over the quantized symbol alphabet [23]. Given a symbol sequence, we estimate the empirical probability mass function and encode the sequence with an rANS stack coder. In addition to the compressed symbol stream, the encoder stores the associated entropy metadata required for deterministic decoding.

**Figure 3:**
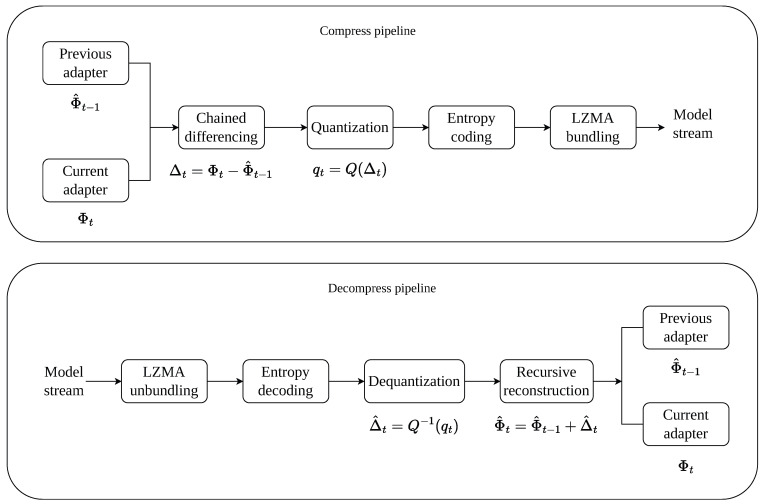
Model-side compression pipeline in SASVC. Scene-specific adapter states are encoded through chained differencing, fixed-precision quantization, rANS entropy coding, and LZMA bundling. The decoder recursively reconstructs the adapter state from the compressed delta stream.

After entropy coding, the resulting binary payload is optionally compressed again using LZMA packaging, producing a compact bundle for deployment and storage. This two-stage design combines fast symbol-level entropy coding with practical file-level archival compression. The complete model-side compression pipeline is summarized in Figure 3.

Overall, the total bitrate is

(12) $$R_{\mathrm{total}}=R_{\mathrm{content}}+R_{\mathrm{model}},$$

where $R_{\mathrm{content}}$ is the bitrate of the LR video stream and $R_{\mathrm{model}}$ is the bitrate of the compressed adapter updates. SASVC improves coding efficiency by reducing both terms jointly: the content stream benefits from downsampling and mature codec engineering, while the model stream is constrained through PEFT and structured compression [4,17,18,19].

### 3.5. Complexity Discussion

SASVC is designed for offline encoding and efficient decoding. The encoder performs scene boundary detection, per-segment adapter optimization, and model-stream compression, all of which are more computationally intensive than decoder-side inference. This design is suitable for practical scenarios where videos are available before delivery, such as video-on-demand, archival compression, or curated streaming pipelines, which is also the deployment setting targeted by prior codec-augmented SR systems [16]. The same design also implies a limitation: because the method relies on per-video scene-specific optimization, it is less suitable for strict live-streaming settings that do not permit encoder-side customization.

At the decoder, only LR video decoding and lightweight SR inference are required. Since the TSR backbone remains fixed and only compact adapters are applied, the reconstruction overhead remains low. Moreover, the use of a low-resolution feature-processing backbone with a final pixel-shuffle upsampler further reduces the computational burden compared with generic high-capacity SR models [14,25]. As a result, the proposed design enables real-time decoding in our experiments while still preserving the gains of scene-specific specialization.

## 4. Results and Discussion

### 4.1. Experimental Setup

**Datasets.** We evaluate SASVC on both long-form and short-form video datasets. This differs from SRVC [16], which primarily emphasizes long videos due to its fixed-interval adaptation scheme. In contrast, our scene-adaptive design is effective for both long and short sequences because model updates are triggered by content transitions rather than by a fixed temporal schedule. For short-form evaluation, we include the widely used UVG [37] and MCL-JCV [38] benchmark datasets. For long-form evaluation, we use 10 longer videos from Vimeo and 5 longer videos from Xiph, and normalize them to 1080p resolution. Together, these datasets cover both short benchmark clips and longer sequences with more irregular scene structure, motion, and texture diversity. All source videos are converted to raw HR frames and then downsampled to generate LR inputs for the content stream.

**Content stream generation.** For each HR sequence, we generate the content stream by spatially downsampling the video and encoding the LR frames using H.265/HEVC [4]. In our main setting, the HR frames are 1080p and the LR frames are 480p, corresponding to a ×4 SR reconstruction setting. We vary the codec operating point using different compression levels to obtain multiple bitrate points for rate–distortion evaluation. Unless otherwise noted, all codec-augmented baselines use the same LR/HR protocol, codec family, output resolution, and evaluation metrics so that the main comparison isolates the reconstruction strategy rather than the content-stream setup.

**Baselines.** We compare SASVC against conventional codec baselines, interpolation-based low-resolution coding, generic super-resolution reconstruction, SRVC-style fixed-segment adaptation [16], and learned video compression baselines such as DVC [7]. For generic SR reconstruction, we include H.265 480p + EDSR [14] as a widely recognized generic SR baseline. We note that stronger generic video-SR baselines such as BasicVSR and transformer-based restorers are also relevant points of comparison; a full matched RD evaluation with these heavier models is important future work. Since TSR is the shared backbone used by SASVC, the proposed method can also be interpreted as adding scene-specific specialization and compressed model transmission on top of a fixed generic TSR reconstructor.

**Model and training protocol.** We use TSR [36] as the shared SR backbone and insert lightweight bottleneck adapters after each major feature block and before the final upsampling stage. During scene-specific specialization, only adapter parameters are updated while the TSR backbone remains fixed. The training objective is the Y-channel $l_{1}$ reconstruction loss, and adapter states are optimized sequentially across scene segments, where the adapter for the current segment is initialized from the previous segment. This warm-start strategy improves convergence and temporal continuity. The segment-wise optimization settings are kept fixed across the main experiments to improve reproducibility, and the key schedule is the same across datasets unless otherwise noted.

**Model-stream compression.** To compress the model stream, we encode chained adapter differences between adjacent reconstructed adapter states, quantize the resulting deltas with fixed 8-bit quantization, and then apply rANS entropy coding [23]. The compressed payload is further packaged using LZMA for storage and transmission convenience. We compare this design against uncompressed weights and first-order independent differencing in the ablation studies.

**Metrics.** We report PSNR, SSIM, and VMAF as reconstruction quality metrics. VMAF is used to better capture perceptual fidelity and practical viewing quality. For bitrate, we report either bits-per-pixel (bpp) or bitrate (kbps), where the total bitrate includes both the content stream and the model stream. We additionally report BD-rate against selected baselines to summarize RD performance over multiple operating points. Unless otherwise stated, the paper focuses on aggregate RD trends across videos; a more detailed study of per-video variance and confidence intervals is left to future work.

### 4.2. Main Rate–Distortion Comparison

**Figure 4:**
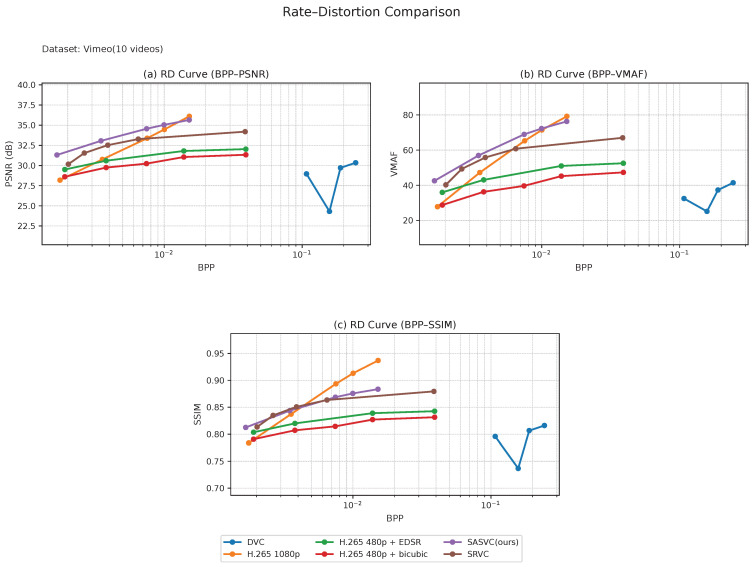
Combined rate–distortion (RD) comparison on Vimeo (10 videos). SASVC consistently provides a better bitrate–quality trade-off than the compared baselines across peak signal-to-noise ratio (PSNR), Video Multi-Method Assessment Fusion (VMAF), and structural similarity index measure (SSIM) views.

**Figure 5:**
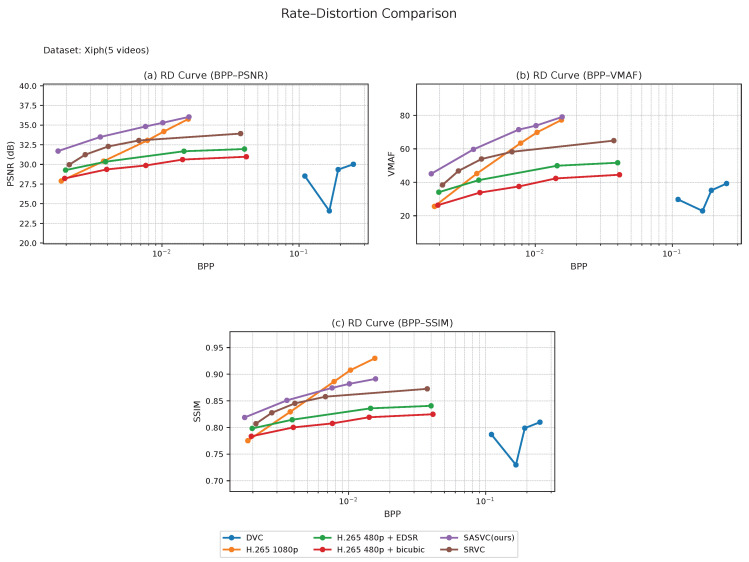
Combined RD comparison on Xiph (5 videos). The advantage of SASVC remains stable on long-form content with diverse scene structures.

**Figure 6:**
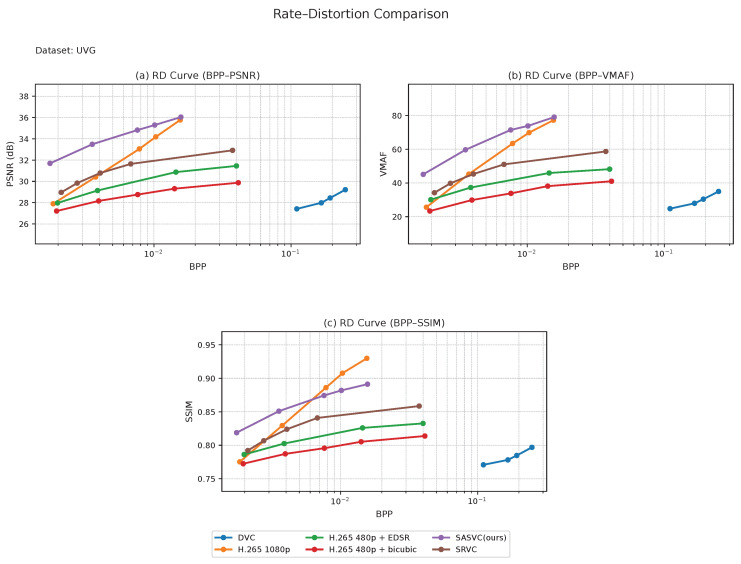
Combined RD comparison on UVG. SASVC remains effective on short-form benchmark sequences despite the reduced video duration.

**Figure 7:**
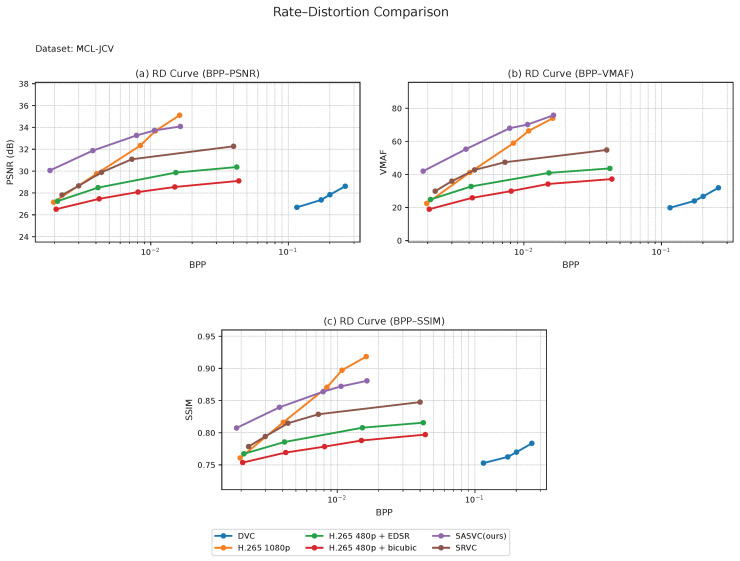
Combined RD comparison on MCL-JCV. The proposed scene-adaptive formulation preserves its advantage on short-form content with varied motion and texture characteristics.

We first evaluate SASVC on the long-form datasets, i.e., Vimeo and Xiph, and then examine its behavior on the short-form datasets, i.e., UVG and MCL-JCV. Across all four datasets, SASVC consistently achieves a more favorable rate–distortion trade-off than both conventional codec baselines and SRVC-style fixed-segment adaptation, confirming that scene-aligned specialization improves the effectiveness of SR-assisted compression under diverse temporal conditions. Although the cross-dataset BD-rate summary below is reported under the PSNR criterion, the RD curves in Figure 4, Figure 5, Figure 6 and Figure 7 additionally show consistent advantages in SSIM and VMAF, which strengthens the perceptual interpretation of the method.

For the long-form datasets, Figure 4 and Figure 5 show that SASVC maintains a stable advantage across a wide range of operating points. On both Vimeo and Xiph, the proposed method consistently outperforms the SRVC-style baseline and remains clearly better than direct H.265 1080p coding in the BD-rate sense. This indicates that scene-adaptive segmentation is particularly effective for longer videos with repeated but non-uniform content transitions, where fixed temporal partitioning tends to introduce unnecessary or suboptimal model updates. Compared with SRVC-style fixed segmentation, the advantage of SASVC becomes especially meaningful when the bitrate budget is limited, suggesting that content-aligned updates improve the utilization of the transmitted model stream.

The long-form results also show that the proposed scene-adaptive design is especially beneficial when scene transitions are irregularly distributed. In such cases, fixed-length chunking may either split a visually coherent segment into multiple updates or merge distinct scenes into a single adaptation interval, both of which reduce the efficiency of the model stream. By contrast, SASVC aligns updates with actual content changes, leading to better use of the available bitrate budget and more stable RD behavior across different quality metrics.

We next evaluate the short-form datasets. Figure 6 and Figure 7 show that SASVC remains effective even when the video duration is limited. This is an important distinction from SRVC-style fixed-segment adaptation, which is primarily advantageous on longer videos. In Figure 6, SASVC still achieves a clear improvement over direct H.265 coding, demonstrating that scene-aligned specialization is beneficial not only for long videos but also for relatively short benchmark sequences.

In Figure 7, SASVC likewise remains superior to H.265 and the compared baselines, although the relative gain is slightly smaller than that on UVG. A likely reason is that MCL-JCV contains richer textures and more diverse spatial details, making high-fidelity reconstruction more challenging under the same lightweight adaptation and bitrate budget. Taken together, the short-form results show that the benefit of SASVC is not tied to long video duration alone but instead comes from improved alignment between model updates and actual visual dynamics.

More importantly, the short-form results reinforce the central claim of this work: scene-adaptive specialization can remain beneficial even when only a limited number of frames are available. This suggests that the proposed framework generalizes well across both temporal scales and content complexities, rather than depending on a specific video length regime.

Across datasets, the advantage of SASVC is most pronounced in the low-to-medium bitrate regime, where unnecessary model updates are especially costly and scene-aligned specialization can more effectively reallocate the available bitrate budget. As the operating point moves to higher bitrates, the relative gain naturally narrows because the codec-preserved content stream already retains more detail and the marginal value of model-side adaptation becomes smaller.

### 4.3. Cross-Dataset BD-Rate Analysis

To summarize the RD advantage over multiple operating points, Table 1 reports the BD-rate (PSNR) results across all datasets. Negative values indicate better rate–distortion efficiency than the H.265 1080p anchor.

SASVC achieves the best BD-rate on all four datasets, with −41.33% on Vimeo, −53.49% on Xiph, −51.53% on UVG, and −39.83% on MCL-JCV. Notably, the gain is consistent across both long-form and short-form settings, indicating that the benefit of the proposed framework comes from improved alignment between model updates and content transitions rather than merely from long video duration. In contrast, the SRVC-style baseline remains beneficial on long-form videos but degrades substantially on short-form datasets, where fixed segmentation can create mismatches between update intervals and actual scene structure.

### 4.4. Ablation Studies

We next analyze the contribution of each component in SASVC. Table 2 summarizes the ablation results under the BD-rate (PSNR) criterion.

Replacing fixed-length partitioning with scene-adaptive segmentation already improves the RD performance by 6.12% while preserving the original throughput. This indicates that content-driven segmentation alone can reduce mismatches between update intervals and actual scene changes. Introducing adapter-based specialization leads to a much larger gain of 58.86%, confirming that scene-specific low-dimensional adaptation is the dominant source of RD improvement. The reason is that adapters let the model adjust high-frequency reconstruction behavior to the current scene while keeping the frozen backbone unchanged, so the update focuses on the content-specific residual that matters most for reconstruction quality. In this sense, scene segmentation and adapters are complementary: segmentation defines *when* the model should change, while adapters determine *how* that change is represented compactly. Finally, adding model-side weight coding further improves the BD-rate to −72.16%, showing that structured compression of the transmitted adapter states is not merely a storage optimization but an important component of the end-to-end compression framework.

The runtime results further show that scene-adaptive segmentation itself introduces negligible decoder-side overhead, while the main throughput change comes from the insertion of adapters. Even with this cost, the final method remains real-time at 125 FPS.

We also examine the role of the segmentation threshold τ. In practice, smaller values of τ produce more frequent updates and a larger model stream, while larger values reduce update frequency but risk merging visually different content into the same segment. Table 3 quantifies this trade-off. In our experiment, τ=20 yields the highest PSNR but also the largest model-stream payload because the video is split into many short segments. Increasing τ reduces the number of segments and the transmitted model size substantially. The operating point used in the main experiments, τ=30, provides a stable compromise between segmentation granularity and model-stream overhead, while the result at τ=35 further shows that performance does not change monotonically with fewer segments. This supports the interpretation of segmentation as an update-scheduling mechanism rather than a semantic scene-understanding module.

### 4.5. Qualitative Analysis of Scene-Adaptive Segmentation

**Figure 8:**
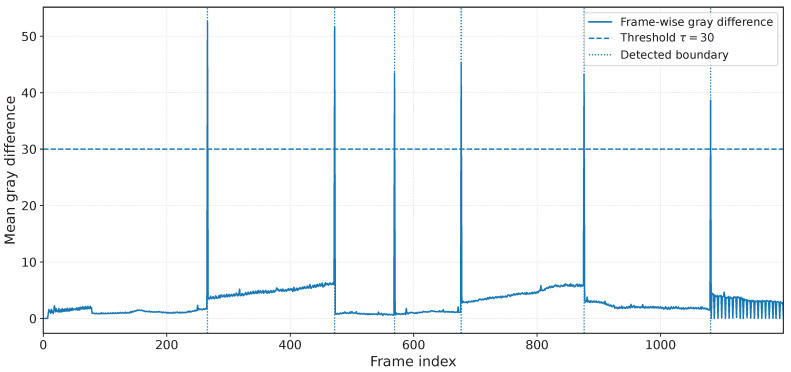
Example of scene-adaptive segmentation on a representative sequence. The frame-wise grayscale-difference signal is plotted together with the threshold τ and the detected scene boundaries. Peaks in the signal trigger model updates, so the resulting partitions follow content transitions rather than uniform temporal windows. This figure is intended to illustrate both the mechanism and the practical meaning of the threshold choice.

Beyond the quantitative ablation, Figure 8 provides a direct visualization of the frame-wise grayscale difference signal used for segmentation. The detected boundaries align with large content transitions rather than with a regular temporal cadence. This behavior is particularly important for practical videos with irregular pacing, where fixed windows can either over-segment stable scenes or merge semantically different content into a single adaptation interval.

**Figure 9:**
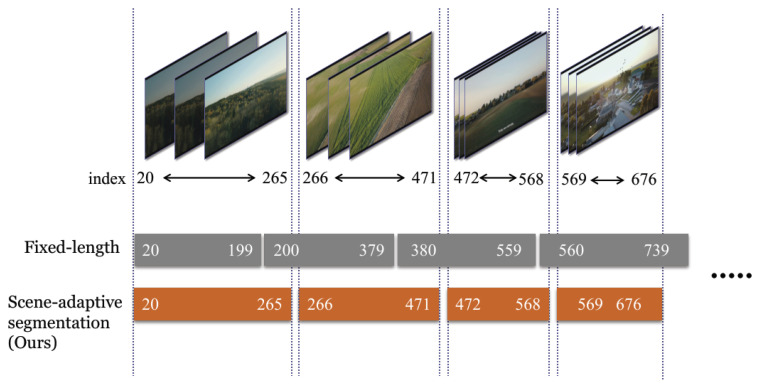
Schematic illustration of scene-adaptive partitioning in SASVC. Unlike fixed-length windows, the proposed approach triggers model updates according to detected content transitions.

For clarity, we also include a schematic illustration of the segmentation mechanism in Figure 9. Together, these two views show both the conceptual design and the actual behavior on real sequences.

### 4.6. Visual Quality Comparison

**Figure 10:**
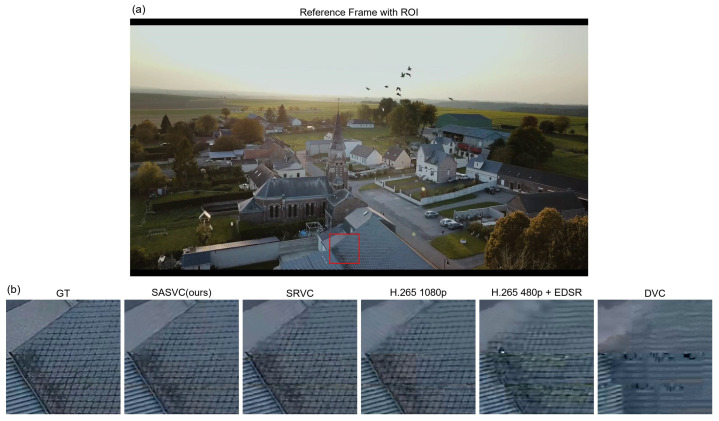
Visual comparison of reconstructed frames with local zoom-ins. (**a**) Reference frame with the region of interest (ROI), where the red box marks the enlarged area. (**b**) Zoomed comparison among GT, SASVC (ours), SRVC, H.265 1080p, H.265 480p + EDSR, and DVC. SASVC recovers sharper edges and more faithful fine structures than direct codec reconstruction, interpolation-based upsampling, and SRVC-style fixed-segment adaptation.

Figure 10 presents representative reconstruction comparisons with local zoom-ins. Compared with direct codec reconstruction and generic low-resolution upsampling pipelines, SASVC recovers sharper edges, more stable fine textures, and fewer local artifacts. Compared with the SRVC-style baseline, SASVC also shows better preservation of scene-specific structures, which is consistent with the benefit of scene-aligned specialization.

These visual improvements are particularly visible around thin structures and high-frequency regions, where fixed or generic reconstruction tends to blur details or introduce unstable patterns. The qualitative results therefore support the RD gains observed in the quantitative evaluation.

### 4.7. Frame-Level Gain Distribution

**Figure 11:**
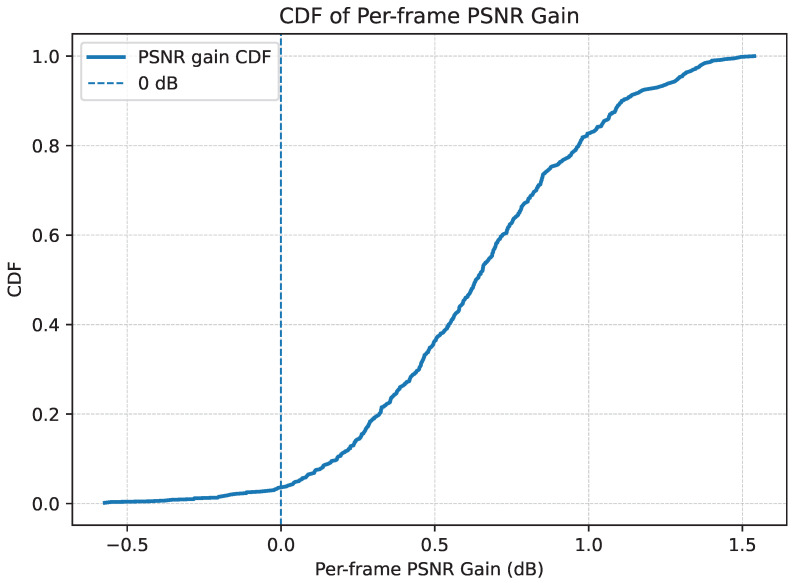
Per-frame gain statistics of SASVC over the baseline. The gain distribution shows that the improvement is spread across the sequence rather than concentrated only near update points.

To verify that the gain is not concentrated only near scene boundaries or update points, we analyze the frame-level improvement distribution. Figure 11 shows the per-frame gain statistics in terms of quality improvement over the baseline. The distribution indicates that the benefit of SASVC is spread across a large portion of the sequence rather than being confined to a small subset of frames.

This observation is important because it suggests that scene-adaptive specialization improves reconstruction quality not only at transition points but also throughout the interior of the detected segments, where the adapted model remains well matched to the local content.

### 4.8. Efficiency Analysis

Decoder-side efficiency is a critical metric for practical deployment. On an NVIDIA RTX 3080 Ti GPU, the proposed method achieves 125 FPS for 1080p reconstruction. Combined with the ablation results in Table 2, this shows that the main RD gains of SASVC do not come at the cost of prohibitive runtime overhead. In particular, scene-adaptive segmentation itself has negligible effect on the inference speed, while the adapter-based specialization remains lightweight enough to preserve real-time decoding.

The efficiency results, together with the RD gains, support the practical design target of SASVC: shift part of the bitrate budget from explicit pixel transmission to compact, scene-aligned model updates, while still keeping the decoder feasible for high-throughput deployment. We emphasize, however, that the current hardware study is limited to the tested RTX 3080 Ti platform; broader benchmarking on CPUs, mobile GPUs, and lower-end devices remains an important deployment-oriented direction for future work.

## 5. Discussion

The experimental results highlight three main observations. First, *scene alignment matters*: even before introducing adapters, replacing fixed temporal windows with content-driven partitioning improves RD efficiency. Second, *compact specialization is effective*: lightweight bottleneck adapters provide a strong improvement over the non-adaptive baseline while maintaining practical throughput. Third, *model-stream optimization is necessary*: once adaptation becomes scene-wise and frequent, explicitly compressing the transmitted adapter states becomes an important part of the end-to-end system rather than an optional engineering refinement.

An especially notable result is the behavior on short-form datasets. While fixed-segment SRVC-style adaptation is primarily advantageous on longer videos, SASVC remains effective on UVG and MCL-JCV because its update schedule is driven by actual content transitions instead of video duration alone. This suggests that scene-aligned specialization may be a more generally applicable paradigm for codec-augmented SR compression.

At the same time, the current design has several limitations. First, the method is aimed at offline compression, so the encoder-side cost of per-video and per-segment adaptation is an application-driven trade-off rather than a free improvement; this is attractive for video-on-demand or archival delivery, but less suitable for strict live streaming. Second, the grayscale-difference segmentation rule is intentionally simple and may fail under fast camera motion, gradual transitions, or abrupt lighting changes, where a content change does not necessarily imply a clean scene boundary. In the present implementation, the frame-difference signal is used directly without temporal smoothing, which keeps the scheduler lightweight but also leaves room for more robust filtered or motion-aware variants. Third, the current runtime results are reported only on the tested RTX 3080 Ti platform, so broader benchmarking on CPUs and lower-end or edge devices remains future work. Finally, the present study focuses on aggregate RD results, and a more complete analysis of per-video variance would further strengthen the empirical picture.

These observations also suggest several specific directions for future work: adaptive thresholding for scene partitioning, motion-aware or learned scene detectors, stronger entropy models for weight coding, broader generic SR/VSR baseline comparisons, and even lighter decoder-side SR backbones tailored to codec-augmented deployment.

## 6. Conclusions

In this work, we presented **SASVC**, a scene-adaptive super-resolution video compression framework that augments a standard codec with a compact, scene-specific neural side stream. The main contributions of the method are threefold: a lightweight scene-aligned update scheduler based on grayscale frame differences, adapter-based scene specialization that bounds the transmitted update size, and a structured model-stream compression pipeline built on chained differencing, quantization, and entropy coding.

Experimental results on both long-form and short-form datasets show that SASVC achieves consistently better rate–distortion performance than SRVC-style fixed-segment baselines and conventional codec settings. In particular, SASVC reaches −41.33%, −53.49%, −51.53%, and −39.83% BD-rate on Vimeo, Xiph, UVG, and MCL-JCV, respectively, under the PSNR criterion. In addition, SASVC maintains real-time decoding at 125 FPS for 1080p reconstruction on an NVIDIA RTX 3080 Ti GPU.

These results suggest that scene-aligned model specialization is a promising direction for neural video compression, especially in offline encoding scenarios where content is available prior to transmission. At the same time, broader runtime studies, stronger generic VSR comparisons, and more robust scene-detection strategies remain important next steps toward wider deployment.

## Figures and Tables

**Table 1:** Cross-dataset Bjontegaard delta rate (BD-rate) comparison under the peak signal-to-noise ratio (PSNR) criterion. Negative values indicate better rate–distortion efficiency than the H.265 1080p anchor.

Dataset	Type	SASVC (Ours)	SRVC	H.265 480p + EDSR	H.265 480p + Bicubic	DVC
Vimeo (10 videos)	Long-form	−41.33%	−17.63%	35.20%	103.04%	5400.27%
Xiph (5 videos)	Long-form	−53.49%	−20.80%	29.75%	103.43%	5688.81%
UVG	Short-form	−51.53%	25.18%	98.75%	278.96%	8836.19%
MCL-JCV	Short-form	−39.83%	26.63%	95.05%	262.93%	8242.31%

**Table 2:** Ablation study of SASVC under the BD-rate (PSNR) criterion. SA, Adp., and WC denote scene-adaptive segmentation, adapter-based specialization, and weight coding, respectively. All values are relative to the fixed-length baseline. Negative values indicate better rate–distortion efficiency.

Method	SA	Adp.	WC	BD-Rate	FPS
Baseline	**✗**	**✗**	**✗**	0.00%	151
+ SA	**✓**	**✗**	**✗**	−6.12%	151
+ Adp.	**✓**	**✓**	**✗**	−58.86%	125
+ Full	**✓**	**✓**	**✓**	−72.16%	125

Checkmarks indicate that the corresponding component is enabled, whereas crosses indicate that it is disabled.

**Table 3:** Sensitivity analysis of the scene-adaptive segmentation threshold τ. Varying τ changes the number of segments and therefore the trade-off between model-stream overhead and rate–distortion performance.

τ	Segments	Model Stream (MB)	PSNR (dB, CRF = 25)	BD-Rate (%)
20	124	82.85	34.8538	−17.94
25	64	42.76	34.8392	−8.15
30	48	32.07	34.8312	0.00
35	44	29.40	34.8244	−4.28

## Data Availability

The data presented in this study are openly available in https://github.com/AdaptiveVC/SRVC (access on 25 April 2026).
